# Prevalence Rates of Loneliness and Its Impact on Lifestyle in the Healthy Population of Madrid, Spain

**DOI:** 10.3390/ijerph17145121

**Published:** 2020-07-15

**Authors:** Daniel Cuesta-Lozano, Leticia Carmen Simón-López, Rubén Mirón-González, Montserrat García-Sastre, Daniel Bonito-Samino, Ángel L. Asenjo-Esteve

**Affiliations:** 1 Department of Nursing and Physiotherapy, University of Alcalá, 28805 Alcalá de Henares, Spain; daniel.cuesta@uah.es (D.C.-L.); leticia.simon@uah.es (L.C.S.-L.); mmontserrat.garcia@uah.es (M.G.-S.); angel.asenjo@uah.es (Á.L.A.-E.); 2 UAH Community Care and Social Determinants of Health Research Group, University of Alcalá, 28805 Alcalá de Henares, Spain; daniel_bonito@fremap.es

**Keywords:** adult, exercise, lifestyle, loneliness, nutrition, observational study, young adult

## Abstract

Background: The Spanish population presents higher levels of loneliness than citizens of countries in Northern Europe. Numerous studies have linked loneliness to increased morbidity and mortality, but very few studies have associated loneliness with healthy lifestyles. The objectives of this research are to identify the feeling of unwanted loneliness in various age and gender groups in the city of Alcalá de Henares (Madrid, Spain), to determine lifestyle habits in the areas of diet and physical exercise, and to examine the association between lifestyle habits and perceived loneliness. Methods: A cross-sectional, observational and analytical study on the perception of loneliness among men (59.06%) and women (60.06%) in a sample (*n* = 611) of the general population (N = 198,945), by means of random assignment of a health survey, was conducted. The data were collected using an ad hoc questionnaire. The data were stratified and analyzed with the IBM SSPS^®^ v.25 software package. Results: The frequency of loneliness is stratified by sex and age, and healthy lifestyle habits in terms of diet and physical exercise are analyzed. Conclusions: People with perceived loneliness do not have worse lifestyle habits. However, women living with other people have a higher perception of loneliness than those living alone. Specifically, the perception of loneliness in young adult women could suggest a low level of moderate physical exercise.

## 1. Introduction

Weiss [1] defines loneliness as a natural phenomenon—a personal feeling that arises at certain times of life and can affect any human being, regardless of gender, age, or other sociodemographic characteristics. We can find different authors who use the concepts of loneliness and social isolation interchangeably. However, the two concepts have different processes. On the one hand, social isolation describes the absence of contact and interactions with the social network, and on the other hand, loneliness refers to subjective feelings of being alone or having the sense that there is nobody around who can help if needed [2]. In sociodemographic terms, women have higher levels of loneliness than men, and it affects young people (15–29 years), seniors (>60 years), and middle-aged adults (30–59 years) [3].

In Spain, loneliness is proportionally higher among senior citizens (>65 years), although in absolute terms, it affects the population aged between 16 and 64 years old to a greater extent, and between 8.5% and 16.1% have some type of affective problem or lack social support. The most heavily affected population is adults living alone, without a partner, and/or with low levels of education and income [4]. This is an important point about loneliness, considering that the Spanish population, as well as the European population, forms an ageing society, with an increasing proportion of people over 65 years old, which is expected to grow by 37.6% by 2033, according to the Spanish National Institute of Statistics (INE) [5].

The main reasons for loneliness in the middle-aged adult population are loss of someone close to them or of a relationship, insufficient social support, and a feeling of inferiority to other people [6]. In addition, the Spanish population has been observed to present higher levels of loneliness than the populations of countries in Northern Europe [7]. Perhaps this is due to the high importance of the family in Spanish culture and society and a greater expectation of maintaining contact with relatives and close friends throughout life.

From the perspective of health, studies on loneliness have focused on ascertaining its relationship with illness. Today, we know that loneliness is related to an increase in mortality and chronic and acute cardiovascular, pulmonary, metabolic, and/or psychological disorders [1]. An association between loneliness and depression has been found, a culmination of the effects of age, gender, education, income, marital status, and social support [8]. Wilson et al. found that the risk of Alzheimer’s disease was more than doubled in lonely people [9]. Physical health can be affected by loneliness too; according to the results of Lauder et al., lonely people were more likely to be smokers and more likely to be overweight or obese [10]. There are also findings indicating associations between high levels of loneliness and hypertension and stroke [11].

In addition, loneliness increases the risk of illness, reduces the probability of acquiring healthy lifestyle habits [12], and increases negative lifestyle habits [3]. Lifestyles can be defined as “the range of an individual’s everyday behavioral patterns and habits” [13]. The origins in the social sciences are thereby included in the socio-medical sciences [14], in order to study the independent and modifiable lifestyle habits that can lead to a change in an individual’s health [15,16,17].

From the perspective of lifestyle and loneliness, we rarely find studies that relate loneliness with lifestyle factors such as sleep [15], physical exercise [16], or nutrition [17]. This evidence gap is expressed by Malcolm et al. [18].

We hypothesize that people who experience perceive loneliness have the worst lifestyle habits. The primary objective is to identify the feeling of unwanted loneliness in various age and gender groups in the city of Alcalá de Henares (Madrid, Spain). The secondary objectives are to determine their lifestyle habits in terms of diet and physical exercise and to examine whether there is an association between the lifestyle habits studied and perceived loneliness.

## 2. Materials and Methods

### 2.1. Study Design

A cross-sectional, observational, and analytical study on the perception of loneliness among men and women in a sample of the general population, by means of random assignment of a health survey, was devised. 

### 2.2. Participants and Setting

The inclusion criteria for the participants were as follows: residents of Alcalá de Henares, at least 18 years of age, with full capacity to answer questions and follow instructions, who agreed to participate in this research study. The exclusion criteria were established by default.

The data were collected by students on the bachelor’s degree course in Nursing at the University of Alcalá. The questionnaire was assigned at random, using a telephone directory, and stratified by districts in the municipality of Alcalá de Henares (N = 198,945 inhabitants). No follow-up of the participants took place.

The sample size was 611 inhabitants, due to protocol compliance, and the participants were aged from 15 to ≥65 years, in Alcalá de Henares (Madrid). The interviews took place between 15 February and 30 April 2018, by telephone, in person, and online. Based on a 95% bilateral confidence interval, at least 580 surveys were considered sufficient to address a 50% indeterminacy, with an error of ± 7.2%.

### 2.3. Variables and Questionnaire

The primary variable of loneliness (dichotomous), and the following secondary variables were collected: living alone (dichotomous), age (divided into 18–29, 30–44, 45–64, and >65 years), gender (dichotomous), degree of loneliness (divided into three ascending levels: does not experience loneliness, some degree of loneliness, and a high degree of loneliness), daily physical exercise, low, moderate, and high intensity physical exercise, daily fruit consumption, daily vegetable consumption, body mass index (BMI) (divided into underweight, normal weight, overweight and obesity), and self-perceived general state of health (divided into very good, good, fair, poor, and very poor).

These variables were collected using an ad hoc, 38-item questionnaire with partially closed questions in Spanish, which was prepared by a panel of experts and divided into the following categories: demographic data, anthropometric parameters, physical activity and exercise, diet, and loneliness.

### 2.4. Legal and Quality Aspects

The guidelines for Strengthening the Reporting of Observational Studies in Epidemiology were followed [19], as well as the Spanish Organic Data Protection Law of 1999 and the Law 14/2007 on Biomedical Research.

### 2.5. Statistical Methods

The data were processed using the IBM SSPS^®^ v.25 software package (IBM Corp. Released 2017. IBM SPSS Statistics for Windows, Version 25.0. Armonk, NY, USA) for protocol compliance. The information was stratified by age and sex. The primary and secondary objectives were examined using frequencies (proportions) and medians (minimum-maximum) when applicable. A non-parameter chi-square test was performed in the analysis of the relationships between the secondary objectives, with a likelihood ratio when applicable, with a binary logistic regression for graded categorical variables, with dummy variables created, and a relationship between discrete variables by a Spearman correlation (rho). The abnormal distribution of the data was confirmed by the Kolmogorov–Smirnov normality test (*p* < 0.05) for *n* > 50. The inferential data are presented as an odds ratio (OR), with a 95% confidence interval (CI), and a significant *p*-value when it was less than 0.05. In multiple comparisons, the threshold of the *p*-value was corrected using the Bonferroni procedure for the family-wise error rate. A Bonferroni adjusted *p*-value < 0.0027 was considered significant.

The secondary variables were expressed according to the European Core Health Indicators [20] and the social determinants according to the INE (Spanish National Institute of Statistics) [21]. The data frequencies were calculated according to various INE reports [22,23].

## 3. Results

The main results are presented in Table 1.

No significant differences were found for perceived loneliness between sex and age (*p* ≥ 0.05). A significantly higher perception of loneliness was found among people living with other people than among those living alone, in women in the 30–44 years age group (OR = 7.5 in 95% CI (1.29–43.41), *p* = 0.017). No statistical significance was observed between the above variables in men in the same age group (*p* ≥ 0.05). No significant differences were found in the other age groups for men or women. No significant differences were found between some degree and a high degree of loneliness among women living with other people, aged 30–44 years old (*p* = 0.133).

In specific terms, the perception of some degree of loneliness when living alone was significantly lower among women aged 30–44 years than the absence of loneliness (OR = 0.026, IC95 (0.002–0.417), *p* = 0.010). Furthermore, no significant differences were found between some degree and a high degree of loneliness among women aged 30–44 who were living alone (*p* = 0.273).

**Figure 1:**
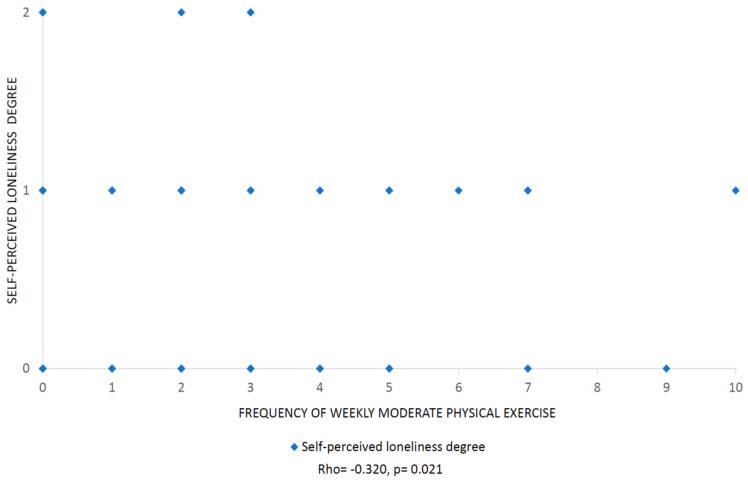
Degree of loneliness in relation to the frequency of moderate physical exercise per week among women aged 30–44 who were living with other people. Degrees of loneliness classified as follows: 0: does not express loneliness; 1: some degree of loneliness; 2: high degree of loneliness. The frequency of moderate physical exercise is expressed as the number of instances of exercise per week. The *p*-value is significant when it is less than 0.05. Abnormal distribution was confirmed by the Kolmogorov–Smirnov test < 0.05 in a sample of (*n* = 52).

No significant differences were obtained for variables in Table 2 related to reported loneliness among women and men aged 30–44 who were living with other people (*p* > 0.0027). As regards the low *p*-value of moderate physical exercise, no significant differences related to more days of moderate physical exercise were found in reported loneliness among women and men aged 30–44 who were living with other people (*p* > 0.05). However, there was a significant inverse correlation between the number of instances of moderate exercise per week and the perceived degrees of loneliness in women aged 30–44 who were living with other people (Figure 1).

## 4. Discussion

### 4.1. Perception and Frequency of Loneliness by Sex and Age

No association between loneliness and the individual’s age or sex was found in this study. This result may be due to the absence of an analysis of social factors due to heterogeneity [1], and it is inconsistent with the majority of observational studies, which report a significant association between increased loneliness and age among individuals aged 18–49 years and over 50 years [7].

As regards gender, significant differences in loneliness in women compared to men have been observed in Spain, of 3.89 ± 1.59 versus 3.56 ± 1.26 *p* < 0.001, at similar levels to those reported in Finland and Poland [7]. We found no significant absence of loneliness in the different age groups in this study or in either sex, but we found a significant increase in perceived loneliness among women between 30 and 44 years old who were living with other people. We cannot compare the results with other studies of the same age range, but an association between loneliness of 3.60 95% CI (3.51–3.69) *p* = 0.018 with the 18–29 age group was found in Rico-Uribe et al. [7].

The frequency of loneliness in all the age groups studied is between 30.30% and 38%, with the perception of some degree of loneliness being more frequent in more than 84%. There is a higher percentage of perceived loneliness in men, of between 86.70% and 100%, compared to women, of between 77.80% and 83.30%. These data differ from a study showing that 90.5% of women had a moderate to high degree of loneliness, with a UCLA score of 49.8 [24]. However, another study obtained a UCLA score of 35.3 with a female participation of 88% [25], which are similar figures to our study and could be considered to indicate a moderate degree of loneliness, since a score of 20 is equivalent to no loneliness and 80 is the maximum.

Most of the studies found focus on loneliness in the elderly population, [24,25,26,27,28,29,30] but our results suggest greater loneliness in adult women between 30 and 44 years old. These results suggest that people perceive loneliness before the onset of any changes due to ageing that enhance loneliness [31].

In the young population (30–44 years), one study finds a significant association between behavioral factors in health and biological and cognitive factors (32–53%) and socioeconomic factors (41–50%) [32]. In this study, we believe that an analysis of the relationship between women between 30 and 44 years old living with other people and the frequency of moderate physical exercise is relevant, and we consider it a potential protective health behavior.

### 4.2. Diet

We know today that loneliness is related to morbidity and mortality [1,32,33], but we do not know the age at which it appears and the lifestyles that influence it. The data that we found suggests that the probability of feelings of loneliness appearing is 7.5 times higher among healthy women living with another person than among those living alone. We found no studies to enable comparison of these results.

We believe that in the healthy adult stage of a human being’s life, habits that have been acquired in previous stages of life persist, and if they are maintained over time, they may lead to illness in the future [24,25,26,27,28,29,30]. This is related to altered eating and exercise habits in the population aged 19–24 years old [34]. In the age group between 15–29 years old in our study, 30% of the men are overweight and 0% obese, and 18.90% of the women are overweight and 5.70% obese. These figures increase in the 30–44 age group, persist in the 45–64 age group, and increase in the group aged 60 years and over. We found no statistically significant differences.

As regards fruit consumption, 56.70% of men and 45.30% of women between 15–29 years consume fruit on a daily basis. The percentages in the group aged between 30 and 44 years old are 63.60% and 55.60%, respectively. With respect to vegetable consumption, 43.30% of men and 39.60% of women between 15 and 29 years old consume vegetables and fruit on a daily basis. The percentages in the group aged between 30 and 44 years old are 45.50% and 38.90%, respectively. These data are related to the idea that consumption habits are established between 15 and 29 years of age and persist into adulthood, which is corroborated by the absence of statistical significance for the association of both variables with the perception of loneliness (*p* > 0.0027).

The data related to the consumption of fruit and vegetables in the age ranges of 45–64 and 65 years and over are similar to the age ranges analyzed. However, it cannot be compared with other studies that also examine loneliness.

### 4.3. Physical Exercise

A study shows that moderate and intense physical exercise reduces mortality due to loneliness by 41% [32]. Despite their knowledge of the benefits of sport, it is apparent that 47% of the population between 19 and 24 years old, who are nursing students, do not engage in any type of physical exercise [34].

The prevalence rates of daily exercise among men and women are the same, at 40% in the 15–29 age group. It is slightly higher in men and somewhat lower in women in the 30–44 age group. In the 45–64 age group, the prevalence rates are similar to the preceding range, and the differences between men and women are smaller. The prevalence rates persist in men ≥ 65 years, and in women it reaches 61%. Moderate percentages for sedentary lifestyles, at between 66.7% and 38.90%, are evident as a consequence, except among women between 30 and 44 years of age, for whom this rate is 72.20%. These data are linked to a decline in moderate exercise in the female age group, which is associated with loneliness, and, as such, a sedentary lifestyle could be considered to increase the risk of loneliness, as reported by some studies [34]. Sedentary lifestyles are related to morbidity and mortality, which can be attributed to biological factors such as high blood pressure and entail a risk of 21% [32]. An association between increased inflammation due to hyperreactivity of the immune system [1] and an alteration in cortisol levels [33] was also observed.

Our study obtained a higher prevalence rate of moderate activity among men than among women in the 15–44 age group, as reported in other studies [34]. For the frequency, we obtained similar prevalence rates for men and women aged 15–29, at 63.33% and 60.20%, respectively, but different prevalence rates in the 30–44 age group, at 63.63% and 22.22%, respectively. These data are interesting as women between 30 and 44 years of age present perceived loneliness at the same rate as those aged 15–29, but the performance of moderate exercise declines by 38%, which does not trigger a significant association with the perception of loneliness when living with at least two other people. However, the frequency of moderate physical exercise per week is related in a protective way to potential situations of perceiving loneliness. Moderate exercise therefore presumably has a relevant risk impact on the perception of loneliness in women aged 30–44 years, which could be caused by biological alterations modulated by social behavior. Given that these are modifiable behaviors which are responsible for avoidable risks, it highlights the importance of this research and justifies the need for future confirmatory studies. Therefore, we consider that this could be a future line of research. The data for intense physical exercise vary between gender and age groups. Levels of physical exercise among men aged 30–44 were 51.82% lower than those in the 15–29 age group, with no statistical significance for perceived loneliness. The data converge with those found in another Spanish study, of 2.7 and 2.5 times a week for men and 2.6 and 2.9 times for women in the 18–54 and 35–54 age groups, respectively [35], and our results would fall within these ranges.

A low prevalence rate of physical exercise at work is observed, at 0.16% and 33.33%, and it is higher among men than women. The amount of physical exercise at work is generally moderate. There are some differences between men and women aged 30–44 in terms of the relationship with loneliness when living with a partner, but it is not significant (*p* < 0.0027). These data are related to the findings in the city of Seville, Spain [16]. In the group aged 15 to 29, 26.66% of men and 17.77% of women perform physical exercise, but these figures do not persist into adulthood. A study found a significant inverse correlation between the level of education and physical exercise at work from 24 to 44 years old [16]. Another study provides significant results in terms of age, sex, relationship with labor resources and physical demands, and academic years in a population aged 50–70 years old [36]. Our data are not comparable as we have no data for education, but an increase in physical exercise at work was obtained among men and women aged 45–64 and ≥ 65 years, perhaps as a result of retirement, although it is not possible to confirm this.

Most studies on loneliness provide information on employment, unemployment, and salary [7,24,27,32]. However, one study examines working conditions in depth, without discussing perceived loneliness [36]. We therefore believe that this may be the first study that relates working conditions with the perception of loneliness.

Therefore, we cannot confirm the hypothesis that people who perceive loneliness have worse lifestyle habits, as no significant differences were obtained. The literature that focused on the gender difference in the relationship between loneliness and lifestyle is scarce. This paper aimed to determine gender differences in lifestyle habits like diet and exercise; it would be interesting to research other lifestyle habits, such as sleep or hygiene, among others. Thereby, more research in this field, also with a qualitative methodology, is needed due to the highly subjective component of the phenomenon of loneliness [1].

### 4.4. Strengths and Weaknesses

The data obtained cannot be extrapolated to society as a whole as the sample size is exploratory. The data are informative and not conclusive.

One of the weaknesses of the study which we identified was a possible response bias due to the non-interventionist design of the study, or the absence of variables such as quality of life, quality of sleep, level of education, financial status, depression, alcoholism, and smoking, which are included in other studies on loneliness [1,7,15,24,26]. Furthermore, no common validated scale was used, as is the case in other studies [32,33], unlike others that do use one [24,25,26,27,28,30]. No qualitative analysis was carried out [29], and as such the loneliness observed this study could be underestimated, as suggested by some studies that find discrepancies between scores obtained with different validated scales [1,33].

Despite the weaknesses, we believe that the sample size and the information provided on social determinants in standardized indices are some of the strengths of this study, which facilitates comparison with other studies. Furthermore, we believe that the study is innovative, as we examine various age groups and healthy people, unlike other studies which focus on illness and older adults [17,25,27,28,30].

## 5. Conclusions

People who perceive loneliness do not have worse lifestyle habits. However, women living with other people have a higher perception of loneliness than those living alone. Specifically, the perception of loneliness in young adult women could suggest a low level of moderate physical exercise. Further research is needed in this field, with larger population samples, in order to seek other possible correlations consistent with these results.

## Figures and Tables

**Table 1:** Description of the sample related to loneliness and lifestyle by age and sex.

**Variables**	**Age Groups**							
	**15–29 (*n* = 274)**		**30–44 (*n* = 90)**		**45–64 (*n* = 150)**		**≥65 (*n* = 97)**	
Female (%)	175 (63.86)		58 (64.44)		91 (60.66)		43 (44.32)	
Self-perceived loneliness (%)	83 (30.30)		29 (32.22)		57 (38.00)		30 (30.92)	
^1^ Some degree of loneliness (%)	70 (84.33)		26 (89.65)		48 (84.21)		25 (83.33)	
^1^ High degree of loneliness (%)	13 (15.66)		03 (10.34)		9 (15.78)		5 (16.66)	
**Stratified according to Perceived Loneliness, Age Group, and Sex**								
	**Man** **(*n* = 30)**	**Woman** **(*n* = 53)**	**Man** **(*n* = 11)**	**Woman** **(*n* = 18)**	**Man** **(*n* = 24)**	**Woman** **(*n* = 33)**	**Man** **(*n* = 12)**	**Women (*n* = 18)**
^1^ Some degree of loneliness (%)	26 (86.70)	44 (83.00)	11 (100.00)	15 (83.30)	21 (87.50)	27 (81.80)	11 (91.70)	14 (77.80)
^1^ High degree of loneliness (%)	04 (13.30)	09 (17.00)	00 (0.00)	03 (16.70)	03 (12.50)	06 (18.20)	01 (08.30)	04 (22.20)
Living with another person	29 (96.70)	51 (96.20)	11 (100.00)	13 (72.20)	22 (91.70)	32 (97.00)	10 (83.30)	10 (55.60)
Underweight	04 (13.30)	06 (11.30)	00 (0.00)	00 (00.00)	00 (00.00)	00 (00.00)	00 (00.00)	00 (00.00)
Normal weight	17 (56.70)	34 (64.20)	04 (36.40)	13 (72.20)	07 (29.20)	22 (66.70)	05 (41.70)	04 (22.20)
Overweight	09 (30.00)	10 (18.90)	06 (54.50)	05 (27.80)	13 (54.20)	09 (27.30)	03 (25.00)	05 (27.80)
Obesity	00 (00.00)	03 (05.70)	01 (09.10)	04 (22.22)	04 (16.70)	02 (06.10)	04 (33.30)	09 (50.00)
^2^ Perceived very good or good health	26 (86.66)	41 (77.35)	09 (81.81)	13 (72.22)	22 (91.66)	29 (87.87)	08 (66.66)	06 (33.33)
^2^ Perceived fair health	04 (13.30)	12 (22.60)	02 (18.20)	05 (27.80)	2 (08.30)	02 (06.10)	03 (25.00)	08 (44.40)
^2^ Perceived poor or very poor health	00 (0.00)	00 (00.00)	00 (0.00)	00 (00.00)	00 (00.00)	02 (06(06)	01 (08.30)	04 (22.22)
**Variables**	**Age Groups**							
	**15–29 (*n* = 274)**		**30–44 (*n* = 90)**		**45–64 (*n* = 150)**		**≥ 65 (*n* = 97)**	
**Stratified according to Perceived Loneliness, Age Group, and Sex**								
**Self-perceived loneliness (%)**	**83 (30.30)**		**29 (32.22)**		**57 (38.00)**		**30 (30.92)**	
	**Man** **(*n* = 30)**	**Woman** **(*n* = 53)**	**Man** **(*n* = 11)**	**Woman** **(*n* = 18)**	**Man** **(*n* = 24)**	**Woman** **(*n* = 33)**	**Man** **(*n* = 12)**	**Woman (*n* = 18)**
^3^ Fruit consumption	17(56.70)	24 (45.30)	07 (63.60)	10 (55.60)	12 (50.00)	19 (57.60)	7 (58.30)	10 (55.60)
Occasions fruit consumed weekly	00 (00–04) ^A^	01 (00–04) ^A^	00 (00–04) ^A^	00 (00–04) ^A^	00 (00–04) ^A^	01 (00–04) ^A^	00 (00–04) ^A^	00 (00–04) ^A^
^4^ Vegetable consumption	13 (43.30)	21 (39.60)	04 (45.50)	07 (38.90)	13 (54.20)	19 (57.60)	04 (33.33)	06 (33.33)
Occasions vegetables consumed weekly	01 (00–04) ^A^	01 (00–04) ^A^	01 (00–02) ^A^	01 (00–02) ^A^	00 (00–04) ^A^	00 (00–04) ^A^	01 (00–03) ^A^	01 (00–03) ^A^
^5.6^ Daily physical exercise	12 (40.00)	24 (45.30)	06 (54.50)	05 (27.80)	12 (50.00)	11 (33.30)	06 (50.00)	11 (61.10)
^7^ Weekly light exercise	26 (86.66)	41 (77.40)	09 (81.81)	12 (66.66)	22 (91.66)	18 (84.84)	10 (83.33)	16 (88.88)
^7^ Occasions light exercise performed weekly	05.50 (00–08)	05 (00–09)	07 (00–08)	04.50 (00–07)	06.50 (00–10)	05 (00–14)	06.50 (01–07)	07 (00–15)
^8^ Weekly moderate exercise	19 (63.33)	33 (60.22)	07 (63.63)	04 (22.22)	13 (54.16)	19 (57.57)	06 (50.00)	10 (55.55)
^8^ Occasions moderate exercise performed weekly	02 (00–05)	02 (00–10)	02 (00–05)	00 (00–04)	01 (00–05)	01 (00–07)	01 (00–06)	02 (00–07)
^9^ Weekly intense exercise	21 (70.00)	22 (41.50)	02 (18.18)	07 (38.88)	05 (20.83)	17 (51.51)	07 (58.33)	06 (33.33)
^9^ Occasions intense exercise performed weekly	02 (00–06)	00 (00–05)	00 (00–07)	00 (00–05)	00 (00–07)	01 (00–07)	01.50 (00–03)	00 (00–05)
Physical effort at work	08 (26.66)	08 (17.77)	03 (27.27)	03 (00.16)	08 (33.33)	09 (27.27)	03 (25.00)	02 (11.11)
^8^ Moderate physical effort at work	06 (00.75)	06 (00.75)	02 (66.66)	01 (33.33)	03 (37.50)	07 (77.77)	02 (66.66)	01 (50.00)
^9^ Intense physical effort at work	02 (25.00)	02 (25.00)	01 (33.33)	02 (66.66)	05 (62.50)	02 (22.22)	01 (33.33)	01 (50.00)

The data are expressed as the number of frequencies (percentages) and as a median (minimum-maximum), as applicable. ^1^ The degree of self-perceived loneliness is graded in categories from lowest to highest: some degree of loneliness and a high degree of loneliness. ^2^ The individual’s self-perceived health is graded in three categories from lowest to highest: very good or good, fair, and poor or very poor. ^3^ Consumption of fruit at least once a day (excluding juice and potatoes) among people (15+ years old). ^4^ Consumption of vegetables at least once a day (excluding juice and potatoes) among people (15+ years old). ^5^ Daily physical exercise, expressed as a proportion of people who perform physical exercise seven times a week. ^6^ Sedentary activities are in the opposite proportion to daily exercise. ^7^ Light physical exercise, expressed as a proportion of people (15+ years) performing physical exercise that does not cause a perceptible increase in the heart rate. ^8^ Moderate physical exercise, expressed as a proportion of people (15+ years) who perform physical exercise that causes a perceptible increase in the heart rate. ^9^ Intense physical exercise, expressed as a proportion of people (15+ years) who perform physical exercise that causes rapid breathing and a substantial increase in heart rate. ^A^ The frequency of consumption is divided into four segments: 00: daily; 01: three or more times a week; 02: once or twice a week; 03: less than once a week; 04: never or almost never. The abnormal distribution of the data was established by the Kolmogorov–Smirnov normality test, with a *p*-value < 0.05.

**Table 2:** Association of factors in women and men aged 30–44 years old who were living with other people, related to loneliness.

Groups	Normality Event in the Loneliness Group/Normality Event in the Non-Loneliness Group	OR CI 95% ^A^	*p*-Value ^B^
Body Mass Index ^1^			
Female loneliness (*n* = 11)/no loneliness (*n* = 19)	09 (69.23)/22 (56.41)	0.516 (0.169–1.575)	0.245
Male loneliness (*n* = 11)/no loneliness (*n* = 19)	04 (36.36) /07 (36.84)	0.812 (0.255–2.585)	0.724
Perceived state of health ^2^			
Female loneliness (*n* = 11)/no loneliness (*n* = 19)	10 (76.92)/36 (92.30)	4.162 (0.772–22.448)	0.097
Male loneliness (*n* = 11)/no loneliness (*n* = 19)	09 (81.81)/16 (84.21)	0.729 (0.120–4.422)	0.731
Fruit consumption ^3^			
Female loneliness (*n* = 11)/no loneliness (*n* = 19)	07 (53.84)/ 27 (69.23)	1.380 (0.841–2.264)	0.203
Male loneliness (*n* = 11)/no loneliness (*n* = 19)	07 (63.63)/10 (52.63)	1.102 (0.540–2.250)	0.789
Vegetable consumption ^4^			
Female loneliness (*n* = 11)/no loneliness (*n* = 19)	05 (38.46)/20 (51.28)	1.029 (0.476–2.226)	0.942
Male loneliness (*n* = 11)/no loneliness (*n* = 19)	05 (45.45)/ 07 (36.84)	0.550 (0.223–1.359)	0.195
Daily physical exercise			
Female loneliness (*n* = 11)/no loneliness (*n* = 19)	05 (38.46)/20 (51.28)	0.354 (0.020–6.307)	0.480
Male loneliness (*n* = 11)/no loneliness (*n* = 19)	06 (54.54)/07 (36.84)	0.193 (0.010–3.880)	0.282
Light physical exercise ^5^			
Female loneliness (*n* = 11)/no loneliness (*n* = 19)	09 (69.23)/36 (92.30)	0.627 (0.130–0.984)	0.109
Male loneliness (*n* = 11)/no loneliness (*n* = 19)	09 (81.81) /16 (84.21)	0.944 (0.562–1.588)	0.829
Moderate physical exercise ^5^			
Female loneliness (*n* = 11)/no loneliness (*n* = 19)	01 (08.33)/15 (38.46)	0.358 (0.462–1.025)	0.046
Male loneliness (*n* = 11)/no loneliness (*n* = 19)	07 (63.63)/12 (63.15)	1.212 (0.701–2.098)	0.491
Intense physical exercise ^5^			
Female loneliness (*n* = 11)/no loneliness (*n* = 19)	04 (30.76)/19 (48.71)	0.771 (0.490–1.214)	0.262
Male loneliness (*n* = 11)/no loneliness (*n* = 19)	02 (18.18)/06 (31.57)	1.063 (0.623–1.814)	0.822
Physical effort at work ^6^			
Female loneliness (*n* = 11)/no loneliness (*n* = 19)	11 (84.61)/30 (76.92)	1.669 (0.843–3.305)	0.141
Male loneliness (*n* = 11)/no loneliness (*n* = 19)	08 (72.72) /14 (73.68)	1.760 (0.722–4.287)	0.214

^A^ Data analyzed by a binary logistic regression. ^B^ The *p*-value is significant when it is less than 0.0027 according to Bonferroni adjustment. ^1^ Normal weight is considered normal for the body mass index. ^2^ The perceived state of health considered normal was very good and good. ^3^ The consumption of fruit considered normal is daily. ^4^ The consumption of vegetables considered normal is daily. ^5^ Light, moderate, and intense physical exercise at least one day a week is considered normal. ^6^ The absence of physical exercise at work is considered normal. Abnormal distribution was confirmed by the Kolmogorov–Smirnov test < 0.05 in a sample of (*n* = 82).

## References

[1] Yanguas J., Pinazo-Henandis S., Tarazona-Santabalbina F.J. (2018). The complexity of loneliness. Acta Bio Med. Atenei Parm..

[2] Gardiner C., Geldenhuys G., Gott M. (2016). Interventions to reduce social isolation and loneliness among older people: An integrative review. Heal. Soc. Care Community.

[3] Richard A., Rohrmann S., Vandeleur C.L., Schmid M., Barth J., Eichholzer M. (2017). Loneliness is adversely associated with physical and mental health and lifestyle factors: Results from a Swiss national survey. PLoS ONE.

[4] Actis W., Pereda C., de Prada M.A. (2004). Salud y estilos de vida en España: Un análisis de los cambios ocurridos en la última década.

[5] (2018). Instituto Nacional de Estadística Proyecciones de Población 2018.

[6] Rokach A. (1989). Antecedents of Loneliness: A Factorial Analysis. J. Psychol..

[7] Rico-Uribe L.A., Caballero F.F., Olaya B., Tobiasz-Adamczyk B., Koskinen S., Leonardi M., Haro J.M., Chatterji S., Ayuso-Mateos J.L., Miret M. (2016). Loneliness, Social Networks, and Health: A Cross-Sectional Study in Three Countries. PLoS ONE.

[8] Cacioppo J.T., Hughes M.E., Waite L.J., Hawkley L.C., Thisted R.A. (2006). Loneliness as a specific risk factor for depressive symptoms: Cross-sectional and longitudinal analyses. Psychol. Aging.

[9] Wilson R.J., Krueger K.R., Arnold S.E., Schneider J., Kelly J.F., Barnes L.L., Tang Y., Bennett D.A. (2007). Loneliness and Risk of Alzheimer Disease. Arch. Gen. Psychiatry.

[10] Lauder W., Mummery K., Jones M.C., Caperchione C. (2006). A comparison of health behaviours in lonely and non-lonely populations. Psychol. Heal. Med..

[11] Cacioppo S., Capitanio J.P., Cacioppo J.T. (2014). Toward a neurology of loneliness. Psychol. Bull..

[12] Yarcheski A., Mahon N.E., Yarcheski T.J., Cannella B.L. (2004). A Meta-Analysis of Predictors of Positive Health Practices. J. Nurs. Sch..

[13] Rodríguez Marín J. (1992). Lifestyle and health. Clínica y Salud.

[14] Coreil J., Levin J.S., Jaco E.G. (1985). Life style--an emergent concept in the sociomedical sciences. Cult. Med. Psychiatry.

[15] Griffin S.C., Mladen S.N., Williams A.B., Dautovich N.D., Lageman S.K., Dzierzewski J.M., Perrin P.B., Rybarczyk B.D. (2020). Sleep Disturbance Mediates the Association Between Loneliness and Health in Older Americans. Int. J. Behav. Med..

[16] García-Fernández J., González-López J.R., Vilches-Arenas A., Lomas-Campos M.D.L.M., Fernández G., López G., Arenas V., Campos L. (2019). Determinants of Physical Activity Performed by Young Adults. Int. J. Environ. Res. Public Heal..

[17] O’Keeffe M., Kelly M., O’Herlihy E., O’Toole P., Kearney P., Timmons S., O’Shea E., Stanton C., Hickson M., Rolland Y. (2019). Potentially modifiable determinants of malnutrition in older adults: A systematic review. Clin. Nutr..

[18] Malcolm M., Frost H., Cowie J. (2019). Loneliness and social isolation causal association with health-related lifestyle risk in older adults: A systematic review and meta-analysis protocol. Syst. Rev..

[19] STROBE Statement: Home. https://www.strobe-statement.org/index.php?id=strobe-home.

[20] European Commission European Core Health Indicators (ECHI). https://ec.europa.eu/health/indicators_data/indicators_es.

[21] (2017). Instituto Nacional de Estadística Encuesta Nacional de Salud. https://www.ine.es/prensa/np770.pdf.

[22] (2018). Instituto Nacional de Estadística Mujeres y hombres en España.

[23] (2008). Instituto Nacional de Estadística Encuesta Nacional de Inmigrantes.

[24] Baek H.S., Won C.W., Choi H.R., Kim B.S. (2007). Loneliness and Cognitive Function in the Elderly Living Alone: Cross-sectional Study. J. Korean Geriatr. Soc..

[25] Petersen J., Thielke S., Austin D., Kaye J. (2015). Phone behaviour and its relationship to loneliness in older adults. Aging Ment. Heal..

[26] Austin J., Dodge H.H., Riley T., Jacobs P.G., Thielke S., Kaye J. (2016). A Smart-Home System to Unobtrusively and Continuously Assess Loneliness in Older Adults. IEEE J. Transl. Eng. Heal. Med..

[27] Fang Y., Chau A.K.C., Fung H.H., Woo J. (2019). Loneliness Shapes the Relationship between Information and Communications Technology Use and Psychological Adjustment among Older Adults. Gerontol..

[28] Machesney D., Wexler S.S., Chen T., Coppola J.F. (2014). Gerontechnology Companion: Virutal pets for dementia patients. Proceedings of the IEEE Long Island Systems, Applications and Technology (LISAT) Conference 2014.

[29] Zamir S., Hennessy C.H., Taylor A.H., Jones R. (2018). Video-calls to reduce loneliness and social isolation within care environments for older people: An implementation study using collaborative action research. BMC Geriatr..

[30] Morris M.E., Adair B., Ozanne E., Kurowski W., Miller K., Pearce A.J., Santamaria N., Long M., Ventura C., Said C. (2014). Smart technologies to enhance social connectedness in older people who live at home. Australas. J. Ageing.

[31] Cardona J., Villamil M., Henao E., Quintero A. (2009). Concept of loneliness, and perception of their current life moment, among elderly adults from Bello, Colombia, 2007. Rev. Fac. Nac. Salud Pública.

[32] Elovainio M., Hakulinen C., Pulkki-Råback L., Virtanen M., Josefsson K., Jokela M., Vahtera J., Kivimaki M. (2017). Contribution of risk factors to excess mortality in isolated and lonely individuals: An analysis of data from the UK Biobank cohort study. Lancet Public Heal..

[33] Lai C.L.J., Leung M.O.Y., Lee D.Y.H., Lam Y.W., Berning K. (2018). Loneliness and Diurnal Salivary Cortisol in Emerging Adults. Int. J. Mol. Sci..

[34] Sánchez-Ojeda M.A., De Luna-Bertos E. (2015). Healthy lifestyles of the university population. Nutrición Hospitalaria.

[35] Elizondo-Armendáriz J.J., Guillen-Grima F., Aguinaga-Ontoso I. (2005). Prevalencia de actividad física y su relación con variables sociodemográficas y estilos de vida en la población de 18 a 65 años de Pamplona. Revista Española de Salud Pública.

[36] Schmitz L., McCluney C.L., Sonnega A., Hicken M.T. (2019). Interpreting Subjective and Objective Measures of Job Resources: The Importance of Sociodemographic Context. Int. J. Environ. Res. Public Heal..

